# Medical Opinion and Movement

**Published:** 1907-07-27

**Authors:** 


### Medical Opinion and Movement.

The seventy-fifth annual meeting of the British Medical Association will take place at Exeter from July 30 to August 3, and, judging from the programme and the names of those taking active parts in the work of the meeting, we are promised some very instructive and fruitful discussions and papers in the various sections. Dr. Henry Davy, President of the Association for 1907-8, will deliver his address on July 30, and on the following day the address in medicine will be given by Dr. Hale White, on "A Plea for Accuracy of Thought in Medicine"; and on Thursday Mr. H. T. Butlin will deliver the address in surgery "Contagion of cancer in human beings, auto-inoculation." Among the subjects arranged for discussion we would notice specially "The indications for operations in cases of intracranial tumour," to be introduced by Dr. J. S. Risien Russell; "Rheumatoid arthritis and the morbid conditions which simulate it," by Dr. H. P. Luff; "Acute nephritis and its results," by Dr. A. F. Voelcker; Dr. A. W. Sykes, and many others. "The relative value of inhalation and injection methods of inducing anæsthesia," to be introduced by Mr. H. P. Dean. The popular lecture will be given by Sir John William Moore, of Dublin, on "Weather, climate, and health," illustrated by screen and lantern slides. An extensive social programme has also been arranged.

The article which has recently appeared in our contemporary *Nature*, from the pen of Professor Donald Ross, must come as somewhat of a shock to those who readily accepted Mr. Morley's assurances in the House of Commons that everything possible was being done by the administrative authorities in India to investigate the etiological conditions of the plague and cope with its ravages. Professor Ross's article is a serious indictment against the civil and military authorities for their utter failure to support those who are ready to pursue researches on the diseases of the tropical climes. According to Professor Ross, there are hundreds of able men with abundant leisure among the Army Medical Staff, who with a little encouragement from the authorities might by this time have succeeded in discovering means of preventing scourges like plague and malaria. But, to quote his own words, "The pigeon-holed report and the official snub awaited us, and we returned disappointed to our idleness." He relates how, as officer of the Indian Medical Service, he once asked his chief if he would like him to investigate fevers during his abundant leisure, and the answer given was that his duty was not to investigate, but to cure! Although Laveran discovered the cause of malaria in 1880, nothing was done in India for 15 years, and even the necessary microscopes were not provided in the hospitals. Such statements by one who is fully competent to speak on the subject cannot but shake our faith in the recent utterances of the Secretary of State for India.

Dr. Carlo Forlanini, Professor of Clinical Medicine in the University of Pavia, has instituted a novel method of treatment for tuberculosis of the lungs. His treatment is based upon the elementary and essential principle of all therapy for any diseased organ of the body—namely, rest. He points out that the characteristic cavities of a phthisical lung are the result of other causes than those consequent on the bacillus of Koch, and may be the outcome of all those micro-organisms which can produce catarrh in the interior of the pulmonary alveoli. This catarrh under the pressure of air during respiration fills the alveolus and impedes circulation, causing denutrition of the tissues of the alveolar walls. In this way the alveolar walls are destroyed, and a small vomica is formed, which by a similar process is enlarged into a cavity of greater and greater dimensions. Dr. Forlanini contends that a lung in process of active destruction cannot get well unless complete immobility can be induced. In support of this view he cites the fact that a pleuritic effusion rendering the lung immobile will cause the lung to heal, although it may lose its functional power. His method of treatment is therefore to render the lung immobile by injecting nitrogen into the pleuritic cavity. The pressure of the gas is so regulated as to be a little higher than the atmospheric pressure, and so overcome the tendency of the lung to expand. The gas, of course, tends to gradual absorption, and the injections therefore must be renewed, the whole treatment lasting several months. Dr. Forlanini has brought forth a good deal of clinical evidence in support of the efficacy of this method of treatment.

Dr. R. T. Williamson, of Victoria University, Manchester, has recently called attention to the "vibrating sensation" as a useful clinical sign in diseases of the nervous system. This "vibrating sensation," or "pallæsthesia," which is determined by placing the foot of a vibrating tuning-fork over subcutaneous bony prominences or surfaces has not received much consideration in this country, but has been studied more fully by observers on the Continent. Various kinds of tuning-forks have been devised for the purpose, but Dr. Williamson uses an ordinary tuning-fork ($C^1$), 9 inches long with prongs $5\frac{1}{2}$ inches long. Vibrating sensations are produced in healthy individuals when the foot of the tuning-fork is placed on any such bony prominences as the styloid process of the ulna, the malleoli, the sternum, and many others. In cases of diseases of the nervous system the author finds that the vibrating sensation is a delicate test for detecting slight impairment of sensation. This sensation may be absent when other forms of sensation are normal or only slightly impaired, as in cases of early tabes, slight peripheral neuritis, and diabetes mellitus. In cases of hemianæsthesia Dr. Williamson finds that this sign is of value in determining between an organic lesion and a functional condition or malingering. In the former case the vibrating sensation is felt when the tuning-fork is placed even on the edge of the sternum of the anæsthetic side, owing to the transmission of the vibrations across the sternum. If, however, the condition be functional, the vibrating sensation will be absent on the anæsthetic side of the sternum.

